# Supplementary material for: Near-complete chiral selection in rotational quantum states
Source: Nat Commun. 2024 Aug 28;15:7441. doi: 10.1038/s41467-024-51360-3 (PMC11358380; doi:10.1038/s41467-024-51360-3)
Supplement: Supplementary file 1 — Supplementary Information [file 41467_2024_51360_MOESM1_ESM.pdf]

Supplementary Information for

Near-complete chiral selection in rotational quantum states

JuHyeon Lee[1], Elahe Abdiha[1], Boris G. Sartakov[1], Gerard Meijer[1], Sandra Eibenberger-Arias[1]*

[1]Fritz-Haber-Institut der Max-Planck-Gesellschaft, Berlin, 14195, Germany.

*Corresponding author. Email: eibenberger@fhi-berlin.mpg.de

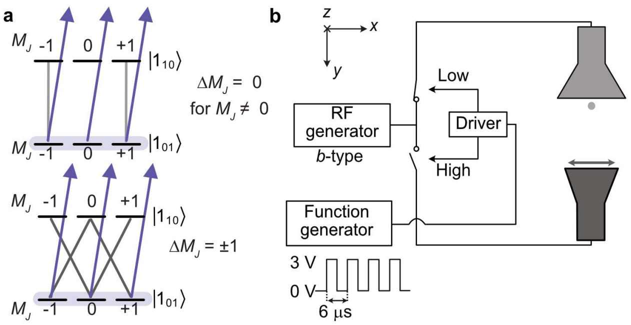

Supplementary Figure 1. Scheme of the experimental procedure and MW setup for the two-level depletion. a, Illustration of the excitation scheme including M_J sub-levels. The first polarization of the MW field along the **z**-axis enables $\Delta M_J = 0$ transitions between the levels with $M_J = \pm 1$ (top), while the subsequent polarization along the **x**-axis enables $\Delta M_J = \pm 1$ transitions (bottom). **b**, Diagram of the MW setup used in the two-level depletion scheme. A MW field, produced by an RF generator, is directed alternatingly to one of two MW horn antennas using a switch. A square wave burst, provided by a function generator to the driver, enables toggling of the microwaves between the antennas with a 6 μs period.

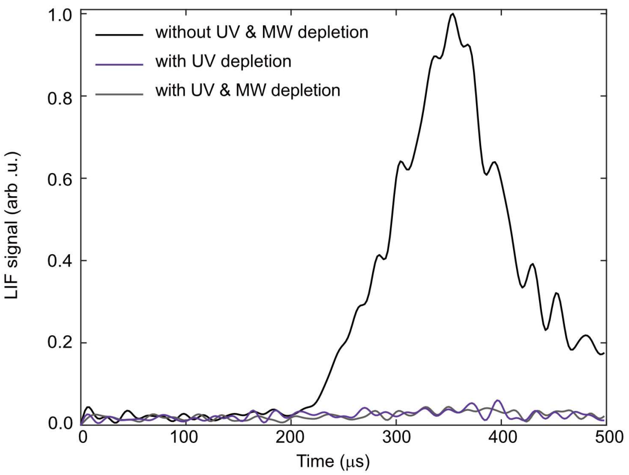

Supplementary Figure 2. LIF signal probing the population in the target rotational state $|1_{01}\rangle$ under various conditions. The black line shows the LIF signal measured without depletion, representing the thermal population of the target state. The purple line depicts the LIF signal, recorded with the UV laser in the depletion region, representing the population present in the target state after depletion. To assess the population in the state $|1_{10}\rangle$, a π-pulse of the MW field driving the *b*-type transition is applied in the ESST region to swap the population between the states $|1_{01}\rangle$ and $|1_{10}\rangle$. The grey line illustrates the LIF signal measured with MW-UV double resonance in the depletion region and the additional MW π-pulse in the ESST region, representing the population present in the state $|1_{10}\rangle$ after depletion. Source data are provided as a Source Data file.

Supplementary Note 1: MW pulse control in the ESST region

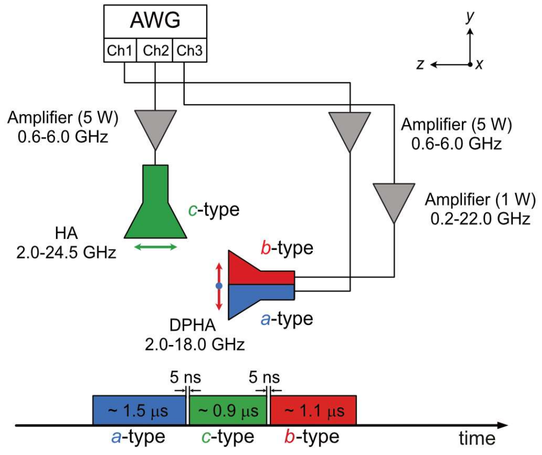

Supplementary Figure 3. Schematic of the MW setup used in the ESST region. The setup includes one horn antenna (HA) and one dual-polarization horn antenna (DPHA) to generate three linearly and mutually orthogonally polarized MW fields. The HA covers a frequency range from 2 to 24.5 GHz, while the DPHA is specified from 2 to 18 GHz. Each MW field is generated from a distinct channel (Ch) of an arbitrary waveform generator (AWG). The MW fields are then amplified and transmitted into the vacuum chamber via the horn antennas. The double arrows and a dot next to the horn antennas indicate the polarization direction of the MW fields. The timing of the MW pulse sequence is illustrated at the bottom of the figure.

In the ESST region, one horn antenna (HA) and one dual-polarization horn antenna (DPHA) are used to generate three linearly and mutually orthogonally polarized MW fields, as shown in Supplementary Fig. 3. To maintain phase stability, all MW pulses are generated using an arbitrary waveform generator (AWG, Keysight M8195A), capable of producing MW pulses with precise phase, amplitude, and frequency up to 25 GHz. Each pulse is created from a separate channel of the AWG, amplified, and then transmitted into vacuum via the horn antennas. Note that while the DPHA is specified to cover frequencies from 2 to 18 GHz, it can be used for the b-type transition at 1.5 GHz. These MW fields are applied consecutively in the following order: $|1_{01}\rangle \xleftarrow{\pi/2,\phi_a} |0_{00}\rangle \xrightarrow{\pi,\phi_c} |1_{10}\rangle \xrightarrow{\pi/2,\phi_b} |1_{01}\rangle$, where ϕ_i represents the phase of the MW field driving the i-type transition, with $i = a, b$ or c. To avoid overlap, there is a 5 ns delay between the end of the first (second) MW pulse and the beginning of the second (third) MW pulse, as illustrated at the bottom

of Supplementary Fig. 3. The frequency range and gain of each electronic component and horn antenna are selected to target the simplest triad of 1-indanol. Our configuration ensures that the pulse lengths necessary to achieve a π pulse for each MW field are around 2 μs in our setup, allowing the ESST process to be completed in less than 4 μs.

Supplementary Note 2: In-beam collision analysis

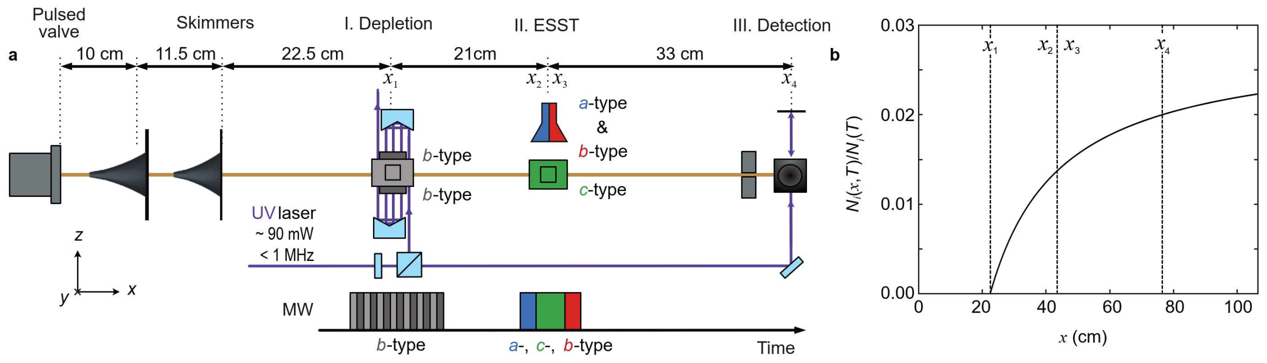

Supplementary Figure 4. Sketch of the experimental setup and calculated re-filling of the population. a, Jet-cooled 1-indanol passes through three interaction regions (I-III). In region (I), a UV laser selectively excites from the target rotational state $|1_{01}\rangle$ to the S_1 electronically excited state, while MW fields driving the b-type transition couple the rotational states $|1_{01}\rangle$ and $|1_{10}\rangle$. In region (II), a sequence of three mutually orthogonal, linearly polarized MW fields is applied for ESST. In region (III), the population of the target rotational state is probed using the same UV laser as in (I). At the bottom, a sketch of the time sequence of the MW fields is displayed. **b**, Re-filling of the population in a previously depleted rotational state is calculated as a function of the position x. Here, x_1 is the location of the depletion region, x_2 and x_3 are the beginning and end of the ESST process, and x_4 is the location of the detection region. Source data are provided as a Source Data file.

The population present in states $|1_{01}\rangle$ and $|1_{10}\rangle$ prior to ESST is the only limitation for achieving 100% enantiomer-selectivity with this experimental approach. Therefore, accurately accounting for these in-beam collisions is crucial for data analysis. A model for the treatment of in-beam collisions in ESST has been previously discussed and is used in our present analysis[1].

The trajectory of the molecular beam is defined along the **x**-axis, with key positions marked as following: x_1 represents the depletion region (I), x_2 and x_3 indicate the starting and ending points of the ESST process (II), and x_4 corresponds to the LIF detection region (III), as depicted in Supplementary Fig. 4a. Given that the total interaction time of the molecules with the MW fields during ESST is less than 4 μs, x_2 and x_3 are considered to be at the same position. The number of molecules in the rotational state $|i\rangle$, with $|i\rangle = |0_{00}\rangle$, $|1_{01}\rangle$, or $|1_{10}\rangle$, denoted as $N_i(x,T)$, varies depending on the position x along the beam and the temperature

T at which the molecules are being thermalized. The rate of in-beam collisions, $dN_i(x,T)/dx$, is proportional to the difference between the thermal population $N_i(T)$ and the actual population $N_i(x,T)$, and to the density of the carrier gas atoms at a given position x. It is approximated by:

$$\frac{dN_i(x,T)}{dx} = \frac{A}{x^2}[N_i(T) - N_i(x,T)], \tag{1}$$

where A is a constant related to the collision cross-section. Solving this differential equation, the population of the rotational state $|i\rangle$ at a given position x can be described as

$$N_i(x,T) = N_i(T) + Be^{A/x}, \tag{2}$$

where B is a constant that can be determined from the initial conditions.

It is assumed that both rotational states $|1_{01}\rangle$ and $|1_{10}\rangle$ are empty at the end of the depletion region, and these are seen to be refilled to ~2% of their thermal populations in the detection region. This enables the calculation of the population in both states (normalized to the thermal population) as a function of x, as depicted in Supplementary Fig. 4b. Their population is thus found to be ~1.4% upon entering the ESST region.

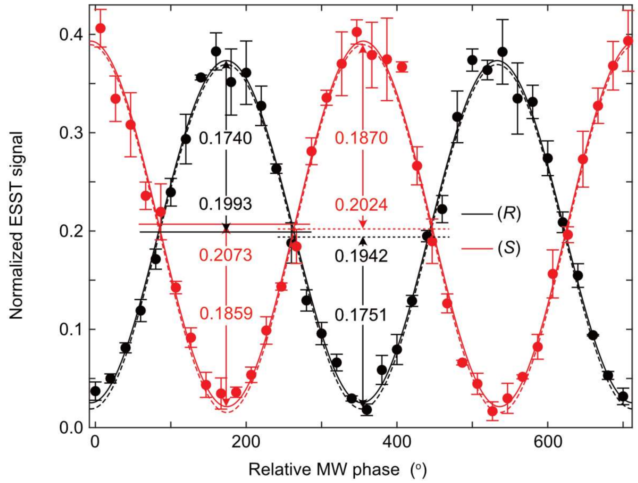

Supplementary Figure 5. ESST results. Normalized ESST signal as a function of the relative MW phase for (*R*)-1-indanol (black) and (*S*)-1-indanol (red). The data points represent the normalized ESST signal measured at x_4, with corresponding sinusoidal fits shown as solid lines. The standard error for each measurement point is indicated by error bars. After accounting for the influence of the in-beam collisions, the ESST signal at x_3 is retrieved, and its

sinusoidal fit is depicted with dashed lines. The amplitudes and mean values for each curve are shown in black for the (*R*)-enantiomer and in red for the (*S*)-enantiomer. Source data are provided as a Source Data file.

In-beam collisions also refill the population in the target state as the molecules travel from x_3 to x_4, thus affecting the ESST signal. To correct for this, the model for in-beam collisions is applied to the measured population in the target state at x_4 to retrieve the population in the target state at x_3. Supplementary Fig. 5 shows the normalized ESST signal measured at x_4 (data points and solid lines) and the corrected normalized ESST signal at x_3 (dashed lines) for both enantiomers. The amplitudes and mean values from sinusoidal fits to the data are given. The comparison shows that the in-beam collisions have a larger impact on the mean values than on the amplitudes, and lead to a decrease of about 3% in state-specific enantiomeric enrichment while traveling from x_3 to x_4; re-filling of the target rotational state due to in-beam collisions is independent of the molecule's handedness.

Supplementary Note 3: Rabi oscillations

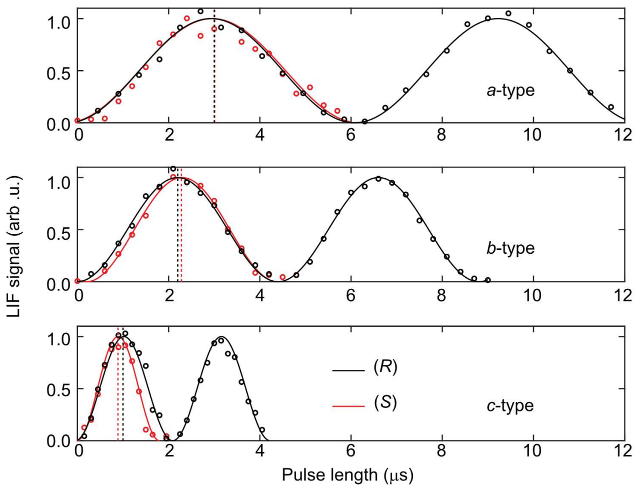

Supplementary Figure 6. Rabi oscillation curves for all three MW transitions of the triad. The black and red curves show the Rabi oscillation curves for (*R*)-1-indanol and (*S*)-1-indanol, respectively. The π-pulse durations are marked by dashed vertical lines. Source data are provided as a Source Data file.

Rabi oscillations are the coherent oscillations in the population of quantum states of two-level systems when driven by an external field. These oscillations reflect the periodic exchange of energy between the system and the external field. The efficiency of the ESST technique depends on the precise pulse conditions of the three MW fields. Therefore, accurately determining the π-pulse conditions for each MW transition is crucial for reliable ESST measurements. To determine the π-pulse conditions, Rabi oscillations are measured for all MW transitions of the triad. In the experiment, one of the two rotational states involved in each MW transition is initially depleted using the UV laser in the depletion region and subsequently re-populated by the MW field in the ESST region. While varying the duration of the MW field, the state population is monitored via LIF detection. For the a and b-type transitions, the UV laser is tuned to a transition from the $|1_{01}\rangle$ state, while for the c-type transition, it is tuned to a transition from the $|1_{10}\rangle$ state. Rabi oscillation curves as a function of pulse duration for both enantiomers are presented in Supplementary Fig. 6. To prevent cross-contamination, separate sources with slightly different settings are used for each enantiomer, resulting in slight variations in π-pulse durations, as depicted in Supplementary Fig. 6.

Supplementary Note 4: Time gate of the LIF signal for ESST analysis

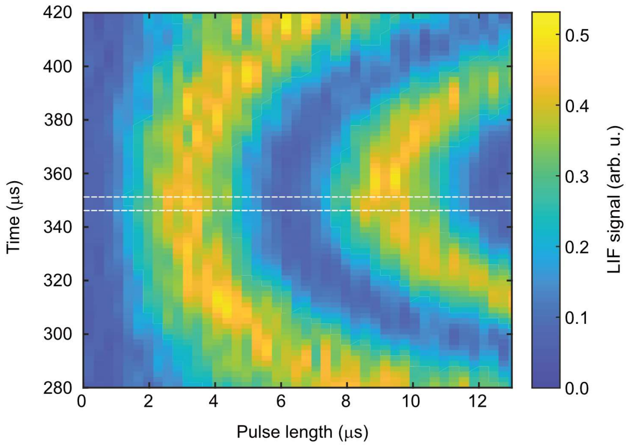

Supplementary Figure 7. Two-dimensional map of the Rabi oscillation measurement for the *a*-type transition. The LIF signal intensity is shown as a function of MW pulse duration (x-axis) and the LIF detection time (y-axis). White dashed lines indicate the 5 μs time gate for the LIF detection, which is used for ESST analysis. Source data are provided as a Source Data file.

Supplementary Fig. 7 presents a two-dimensional map of the Rabi oscillation measurement for the a-type transition, where the LIF signal intensity is plotted against the MW pulse duration and the detection time. The first brighter region corresponds to the π-pulse condition. In the figure, a shift in these π-pulse durations along the LIF detection time axis is evident. This indicates that molecules detected at different times have interacted with different MW field intensities at the ESST region. Therefore, not the entire integrated LIF signal is used for the analysis of ESST. Instead, a portion of the LIF signal within a time interval of approximately 5 μs, indicated by white dashed lines, is used for ESST analysis.

Supplementary Note 5: Confirming that the ESST signals of both enantiomers are exactly out-of-phase

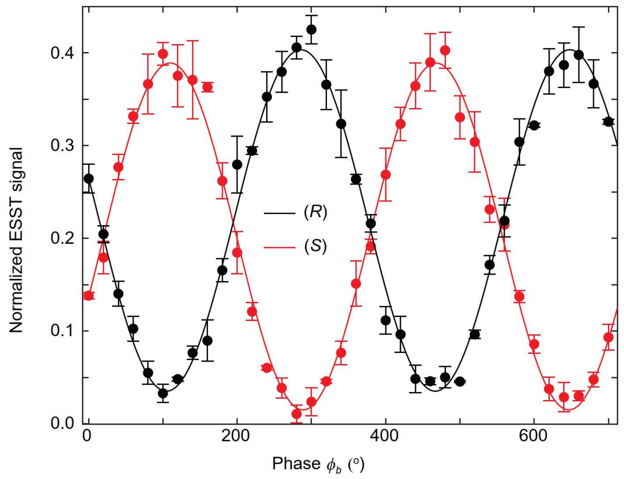

Supplementary Figure 8. ESST results. Normalized ESST signal measured with identical pulse durations as a function of the phase ϕ_b are shown for (*R*)-1-indanol (black) and (*S*)-1-indanol (red). The pulse durations are optimized for the (*S*)-enantiomer. The standard error for each measurement point is indicated by error bars. Source data are provided as a Source Data file.

When using identical pulse durations for both enantiomers, the ESST signal is expected to show a phase difference of π between them. To confirm this, the ESST signals for both enantiomers are measured using pulse durations that are optimized for the (*S*)-enantiomer. Pulse durations of 1.5 μs, 1.14 μs, and 890 ns are applied for the a, b, and c-type transitions, respectively. Supplementary Fig. 8 illustrates the normalized ESST signals as a function of the phase ϕ_b in black for the (*R*)-enantiomer and in red for the (*S*)-enantiomer. As expected, an exact π phase shift is observed between the two curves.

Supplementary References

1 Lee, J. H. *et al.* The influence of microwave pulse conditions on enantiomer-specific state transfer. *New J. Phys.* **26**, 033015 https://doi.org/10.1088/1367-2630/ad2db4 (2024).
